# Obstacles and Future Prospects: Considerations on Health Promotion Activities for Older Workers in Europe

**DOI:** 10.3390/ijerph15061096

**Published:** 2018-05-28

**Authors:** Nicola Magnavita

**Affiliations:** Institute of Public Health, Università Cattolica del Sacro Cuore, 00168 Roma, Italy; nicolamagnavita@gmail.com; Tel.: +39-347-3300367

**Keywords:** health promotion, ageing, workplace, occupational health, effectiveness, salutogenesis, holistic medicine, subsidiarity, participatory approach, setting

## Abstract

The ageing of workers is one of the most important issues for occupational health and safety in Europe. The ageing of the active population means that health promotion is a necessity rather than a mere option. This review considers barriers and perspectives for workplace health promotion for older workers. Lack of awareness on the part of management and inflexibility in the occupational health and safety system appear to be major barriers. To overcome these, it will be necessary to disseminate knowledge regarding the effectiveness of health promotion actions for older workers, encourage greater involvement on the part of social partners, recover resources by replacing medical consumerism and bureaucratic practices, adopt an integrated approach combining the prevention of occupational risks and the promotion of healthy lifestyles, and recognize subsidiarity and the ability of working communities to regulate themselves.

## 1. Introduction

Ageing of the workforce presents a challenge for all European countries. Keeping workers active and productive through health promotion intervention is a prime objective of European labour policy. The general framework of the ProHealth65+ research funded by EU-CHAFEA to examine workplace health promotion for older workers (WHPOW) in 10 countries belonging to Mediterranean (Italy, Greece, Portugal), Western (Germany, Holland, Czech Republic), and Eastern Europe (Hungary, Poland, Bulgaria, Lithuania) [1] was a stimulus to this commentary.

Considerable differences were found in the various types of intervention from country to country and also between different regions of the same European state. The number of intervention studies was very small compared to the number of companies and work sectors involved in the ageing workforce phenomenon. Health promotion activities therefore appeared to be inadequate for the scale of the social and economic changes taking place [2], as can be seen from the results of studies published in detail elsewhere [3,4].

After the previously published quantitative analysis, my aim in this paper is to make a commentary of WHPOW intervention studies that focuses particularly on incentives and the barriers obstructing their implementation. I report my main considerations and put forward proposals for improving health promotion activities.

## 2. Source of Experiences

To identify intervention projects carried out in European countries between 2000 and 2015 we carried out (1) a systematic review of the literature; (2) research on gray literature; and (3) an analysis of major companies using SurveyMonkey. The first stage was a systematic review conducted by searching electronic databases (MEDLINE, ISI Web of science, SCOPUS, The Cochrane Library, CINAHL and PsychINFO) and identifying articles in English published between January 2000 and May 2015 [5]. The second stage was a snowball search on the Internet starting from the most important European Network websites that dealt with this topic in order to identify activities carried out by various institutions in support of older workers. Finally, companies were contacted directly online. The list of companies to be contacted was obtained by combining the rankings of the world’s major corporations according to sales, brand, appreciation by workers, and attention to the older worker [6]. Additional information was obtained by contacting a panel of experts from each of the 10 European countries and, when possible, the authors of the studies. I also extracted key findings regarding the study design, type of company and business sector, subjects and objectives of the retrieved studies. The main objective of the ProHealth65+ research was to identify the institutions active in health promotion for older workers and the role that each had played in the promotion, organization, financing of activities or the provision of rules and expertise for interventions.

The ProHealth65+ project, in its three years of activity, involved 22 coordinating partners and 37 researchers from 12 research institutions located in 10 European countries, plus 14 members of the advisory board, and 18 Institutions acting in the board of health promoters. In each of the European countries, three to 12 experts at national/governmental or scientific/academic or local/company level were contacted. The major European companies were contacted through an online survey and the available managers of around 30 companies provided further information on their health promotion experiences.

The talks conducted during the three years of the project with national experts and some of the people directly involved in the promotion interventions have allowed a comparison of opinions on the main obstacles that health promotion for the older worker encounters in the workplace. The general considerations that emerged and which are confirmed by the literature are the subject of this study. The opinions expressed in this paper should be attributed exclusively to the author and do not reflect either the official position of the ProHealth65+ research group nor that of the companies or other institutions that have participated.

## 3. Obstacles to Health Promotion

More than 80% of the interventions we recorded were carried out in medium-sized companies. Although some projects designed to promote the health of older workers in small companies may not have been reported in the literature and therefore escaped our attention, we believe that WHPOW is less common in small companies than in larger ones, mainly due to a shortage of financial and human resources for implementing intervention. Obstacles encountered in carrying out WHPOW also include a lack of flexibility in organizing tasks (e.g., job rotation) and an uncompromising attitude on the part of employees and managers. Moreover, existing rules do not always lend support to evidence-based practice [7].

We observed that not all European nations produced national guidelines for WHPOW (among those selected, only Bulgaria, Czech Republic, Germany, Lithuania, Netherlands, and Poland), possibly due to less interest in the problem than other countries. Another limiting factor concerned the time taken to implement health promotion measures [8].

A reluctance to change work habits and practices, mostly on the part of long-serving employees, also constituted a potential obstacle. Previous intervention studies showed that the age of workers influences behavior and lifestyle modification and that older workers are more resistant to change than younger ones, for example as regards smoking [9] or vaccination [10]. This may occur due to a lack of awareness of the dangerous effects of some habits or due to poor communication concerning the benefits of better working practices or a healthy work environment. A greater effort should be made to increase the motivation of older workers. This can be achieved by tailoring interventions and involving the workers, especially the more experienced ones, in decision-making. A lack of worker participation in the design and implementation of health promotion activities can also affect worker motivation. Finally, low wages can cause a drastic reduction in the motivation of employees as regards WHP. Group interventions based upon self-management participatory principles have been proposed for improvement of work engagement and work limitations of older workers [11].

### 3.1. Resources and Continuity

In general, a lack of resources was the most frequently reported obstacle for WHP programs, while strong management support was most frequently reported to be the factor that facilitated this type of intervention [12].

Supranational institutions play a very important role in WHPOW as a guide for national governments and as providers of funds. Funds for WHPOW are also made available by many national and local public or private bodies or by the companies themselves. It is usually company management that decides to take WHPOW action if it believes that workforce aging is an important topic for health promotion. However, this does not occur in most companies. A recent study showed that the strategy favored by employers from many European countries (Denmark, Germany, Italy, The Netherlands, Poland, and Sweden) is not the promotion of active aging but the replacement of older with younger and relatively better-educated workers [13].

We must point out that promotion does not mean prevention. Most countries have detailed rules for occupational health surveillance that have significantly improved health in the workplace by reducing accidents, preventing occupational diseases, and optimizing job design and work systems. The omission of such preventive measures in workplaces is severely punished. Occupational health surveillance, if applied to aspects related to work only, cannot in itself be considered health promotion intervention. The International Labor Office has called for broader intervention that combines traditional occupational medicine focused mainly on the prevention of accidents and occupational diseases with health promotion activities. Such WHP programs are designed to help workers to become more skilled in managing their chronic conditions and more proactive in their health-care by improving their lifestyles, the quality of their diet and sleep, and their physical fitness. These issues should be addressed from an individual point of view and from a collective one that is closely related to the improvement of working conditions, the occupational environment, work organization, and family, community, and social contexts [14]. These interventions overcome the traditional distinction between professional and non-professional risk factors.

We can observe that prevention of work-related risks is an obligation for the employer, health promotion is not. The latter, however, is of particular interest to society. Ageing of the workforce has such important repercussions on the productive capacity of public and private enterprises that health promotion for older workers has become a vital issue. Some European countries have already taken important steps to tackle this question. Supranational institutions are urging all European countries to rapidly adopt the same correct approach.

The topic of how the government can modify risky habits and lifestyles is much debated in literature. According to some researchers, new approaches to evaluating population-based interventions, such as taxes and regulation, must be implemented [15]. Other scientists observed, on the other hand, that “The political governing systems’ vertical hierarchy has control over money and laws, neither of which materially affect individual lifestyle/behavioral choices toward health” [16]. Therefore, every effort must be made in teaching people to choose autonomously the best solutions in order to achieve a higher level of public’s health. On the contrary, a process exclusively focused on punitive actions (e.g., taxation of unhealthy products or behaviors) will only get people migrating from one unhealthy product/behavior to another without any useful results. In a completely similar way, governments can act on companies through punitive or reward mechanisms. We can think that the latter are more suitable for health promotion interventions that require awareness and commitment from both management and workers.

A successful WHP program produces intangible benefits due to increased worker wellbeing and satisfaction. However, since these benefits are not immediate, an initial investment in WHP projects is always necessary. Projects should explicitly include ways of recovering resources so as to ensure continuity of intervention. Since small companies often lack access to health-promotion opportunities available at larger workplaces, it could be useful to provide incentives for smaller workplaces to implement comprehensive WHP programs [17].

Most European countries already allocate substantial resources for occupational health, but in many cases, these resources are not invested profitably. Although the workplace is an ideal setting for health promotion for older people, it is also the place in which huge resources are being used for purposes other than health promotion. Improving the quality of occupational health in the workplace is the best way to recover resources for health promotion. The true task of occupational medicine is to improve occupational health. This can better be achieved by adopting a holistic approach that takes into account occupational risks, technical and medical expertise, and ergonomic adaptations in the work environment and habits and lifestyles that may favor the onset of diseases and thereby interfere with working capacity.

The holistic approach to occupational health is considered to be of primary importance in some central and northern European countries. The most numerous and effective WHPOW projects are concentrated in Scandinavian and central European countries [18,19]. In these countries, health promotion often has fiscal incentives. In Flanders, for example, the state recognizes company commitment to health promotion and participates by providing some of the necessary funding [20]. This significant commitment to the promotion of occupational health services should act as a guide for policy and labor laws in other European countries.

A particular aspect of health promotion concerns the management of disability. Recovering the full working capacity of a worker with a handicap is certainly better than providing compensation for his/her handicap. Germany offers an excellent example of how workers with disabilities can recuperate without the provision of monetary compensation [21]. This policy is crucial for older workers who often suffer from chronic diseases and disabilities.

Continuity is one of the crucial aspects of WHPOW projects: in many cases a project is sustainable only if it leads to a recovery of resources or a significant improvement in production methods. Many projects come to an end if they are not continuously financed. This general rule has some exceptions. Some Dutch WHPOW projects that were temporarily funded by the government had a positive outcome because several companies decided to take over responsibility for funding after witnessing the beneficial effects of the projects [22].

To be effective, a promotion program requires economic resources and, above all, human resources and scientific knowledge. Health promotion must be based on evidence [23,24,25,26].

### 3.2. Evaluation of the Effectiveness of WHPOW

Authoritative researchers have stated that much is done in workplaces to control hazards and to improve health, but far too little of it is measured for effectiveness [27]. Since data on individual risk factors for chronic diseases (smoking, physical activity, body mass) are collected by company physicians in a variety of ways, it is difficult to make comparisons, evaluations, or carry out research [28]. The evaluation of results remains a crucial issue for WHPOW programs. Most WHPOW programs do not envisage an evaluation of results, and even when an assessment is made, there is a lack of scientific evidence as evaluation is performed at the end of a project rather than after a period of time when the lasting effects of the improvements achieved can be demonstrated. Furthermore, since WHPOW programs rarely contemplate the existence of an external advisor, there is the risk of a conflict of interests if evaluation is carried out by those conducting the WHPOW intervention. The same applies to other aspects of workplace health promotion. Although some evidence suggests the long-term effectiveness of multicomponent lifestyle interventions in the workplace, the limited number of high quality studies and the lack of consistency among studies preclude a conclusive assessment. More comprehensive, well-reported studies are needed to fully understand the impact of interventions [29,30,31,32,33,34,35,36,37,38,39,40]. Methods such as ROI (Return On Investment) that are used to assess the effectiveness of programs need to be carefully evaluated since their application has led to considerable criticism [41]. Nevertheless, it is widely accepted that well-designed and well-executed intervention studies founded on evidence-based principles can achieve positive health and financial outcomes [42].

### 3.3. Occupational Health: Current and Historical Approaches

The system of insurance against occupational diseases was set up in most developed countries a hundred years ago to deal with industrial work in which occupational hazards were an inevitable consequence of production. Since it was not possible to change the occupational environment, the only form of justice was to offer compensation to the sick worker.

A century later, the situation in more developed countries has changed completely. New technologies have brought more safety to all work environments. A significant decrease in many “traditional” occupational risks, such as exposure to silica, asbestos, toxic chemicals, noise, and vibrations, has been accompanied by the growing importance of new risks such as work-related stress and other psychosocial stressors, repetitive movements, strenuous efforts, and incorrect postures. In spite of these changes, the approach to occupational health in many countries has remained substantially the same as a century ago. Occupational health services still look for early signs of disease, and the insurance system provides compensation for the sick worker. This so-called “laboristic” approach, that focuses only on occupational aspects, fails to take into account the health risks that are not directly related to work in spite of the fact that diseases arising solely from work (e.g., silicosis, lead intoxication, noise deafness) constitute only a minute part of all chronic diseases affecting workers in developed countries. European Directives oblige employers to implement occupational health and safety measures in all countries, and today, most causes of disease are to be found outside the workplace. All the diseases that European workers attribute to work (so called “work-related diseases”) are actually due to a number of factors. Musculoskeletal disorders—by far the most frequent complaint affecting European workers—are the result of several different factors that include heavy load carrying, awkward postures, repetitive movements, lack of exercise, obesity, psychosocial factors, and many others. Exposure to each of these risks may be both occupational and non-occupational. If an employee has back pain, a physician cannot clinically detect the cause of this disorder.

In countries such as Italy, where the relationship between work and health is traditionally seen as an aspect of labor relations, the “laboristic” approach and the possibility of obtaining financial compensation for work-related back pain can lead to the so-called “compensation syndrome,” where the employee emphasizes all work-related aspects of his/her illness and overlooks others. A physician, who in most cases is a trade union physician rather than the company physician, makes a diagnosis of occupational disease. The insurance institute conducts an investigation and, if the occupational cause and effect relationship is established, compensates the employee and punishes the employer, who may be sued for damages by the worker and prosecuted by the state. All this process takes years and does not improve health.

If the current “laboristic” approach were to be integrated with a more holistic approach, as in Scandinavian countries and continental Europe, far more resources would be available to invest in promotion against all health risks. This approach is urgently needed in the case of older workers, since they have the highest level of disability and chronic illness due to both occupational and non-occupational origin. In fact, it is vitally important to adopt a new approach to health promotion in occupational health and safety (OHS) practice [43]. The workplace could become the ideal environment for promoting a healthy lifestyle [44].

### 3.4. Unnecessary Bureaucracy

In some cases, public resources that have been allocated for the health of workers are used in an unprofitable way. For example, every year, the Polish Occupational Medicine Service (OMS) carries out 4.5 million mandatory preventive examinations of individuals who are seeking employment [45]. After this medical examination, a qualified physician issues a certificate stating whether or not the individual is fit for work. A similar procedure has been in force for many decades in Italy. The “healthy and robust constitution” certificate was considered a mandatory pre-employment requirement. Despite the demonstration that this practice was devoid of clinical efficacy [46], it was only recently abolished. Many Italian public bodies, however, continue to request a certificate of “physical fitness” before knowing the type of work to be done and the occupational risks associated with it [47]. It is hard to understand the usefulness of this kind of procedure, which neither prevents occupational risks nor promotes healthy lifestyles.

To have sufficient resources for workplace health promotion, it is essential to avoid this type of unnecessary expense.

### 3.5. Target Health, Not Diseases

Some researchers argue that health promotion interventions should be directed specifically at workers with an elevated risk of chronic disease. A recent, very interesting review selected some US workplace interventions that targeted at-risk employees on the basis of the latter’s disease or disease-related risk factors [48]. However, this approach is not suitable in workplaces where fairness requires health promotion activities to be offered to all workers, not just to a limited number. The ideal solution is to promote health rather than look for disease. The presence of occupational health services in many workplaces allows this to be done.

In Italy, occupational health physicians carry out around 10 million mandatory medical examinations and routine check-ups a year-probably the largest number in Europe. However, only a few of these activities are aimed at health promotion [49].

This large number of annual medical examinations (most of which include blood and urine tests despite their acknowledged low job-specificity) is the result of a “health surveillance” concept that has been distorted by cultural and historical factors [50]. Unless there is a clear diagnostic purpose, it is useless to carry out blood tests or instrumental analyses. These are carried out nevertheless because workers believe that it is their right (“acquired right”) to undergo these tests, and employers consider them to be a more tangible procedure than a health education program. OHS companies also find it easier and more profitable to sell health products rather than set up, implement, and check the long-term results of a health promotion program.

It is debatable whether these medical examinations should be carried out at the expense of the employer and in a country that offers an efficient and universal Public Health System with the screening, diagnosis, and treatment of non-work-related diseases. Clearly, we seem to be over-testing, but are we offering correct and adequate health promotion at the same time?

### 3.6. Linking Industrial Hygiene and Occupational Health

Misuse of health care resources also occurs in current occupational health practice where many occupational physicians face a number of important limitations due to a lack of clinical and occupational exposure information at the time of medical examination, or even owing to threats to their professional independence. This situation often occurs in external OHS services, especially in connection with information regarding occupational and non-occupational sickness absence data, participation in the investigation of work accidents and occupational diseases, and access to the prevention service on the part of workers.

Poor coordination and communication between the OHS, the National Health Service, and the employer’s insurance companies in addition to a failure to exchange medical records when a worker changes jobs or an employer contacts a different external OHS service, impair occupational health practice. The lack of adequate exposure information (e.g., job description, risk evaluation, environmental measurements) gives rise to particular concern because, without the necessary information, Occupational Medicine loses all meaning. The occupational physician has a duty to assess the condition of each individual worker and give advice for improving the working environment by adapting it to the individual.

Existing evidence supports an integrated approach [51]. If industrial hygiene and occupational health function separately, working conditions may be seriously impaired. Unfortunately, in some countries, such as Poland, where the Work Safety and Hygiene service and the Occupational Medicine service are separate [45], integration between services is very rare.

### 3.7. Accessibility of Health Care in the Workplace

Accessibility poses an important problem for external prevention services. Most European enterprises are small or medium-sized and are often distributed over an extensive geographical area. Physicians employed by external occupational services are often located at some distance from workplaces so their contact with workers is limited to routine medical examinations. On the contrary, physicians working for internal prevention services have good accessibility and are able to examine workers more frequently after sick leave, thereby increasing the probability of early detection of occupational health problems and vulnerable workers. An assiduous presence of the medical service is essential for successful health promotion, since recent studies in the field [52] have shown that there is a constant demand for the advice of an occupational physician.

### 3.8. Fitness for Job

The fact that health examinations are almost always followed by a fitness-for-work certificate that is issued regardless of the type of job and its associated risks, may have an unintentionally detrimental effect on workers. Since it is claimed that companies are reluctant to accept workers who are given a “conditional” fit–for-work certificate, some workers who could most benefit from WHP activities may decide to avoid this type of prevention service. Employee participation is an essential requisite for workplace health promotion.

### 3.9. Subsidiarity

Subsidiarity is another significant aspect of the northern European model of health and safety promotion in the workplace. In compliance with this principle, the state rarely intervenes in consultations among social partners. Safety and health at work are primarily the responsibility of companies and workers. The transition from a proto-industrial top-down, authoritarian model to a more modern participatory and democratic bottom-up pattern is not easy, especially in countries where there is a high unemployment rate. However, according to European Directives on Health and Safety at Work founded on worker participation, this is the path that should be undertaken.

Paradoxically, countries like Italy that have a long-standing tradition of occupational medicine find it difficult to follow these indications. The largely inflexible law system that imposes the penal code on the whole field of health and safety at work can become an obstacle to health promotion. An example of this concerns the prevention of sleepiness at work. It is well known that many occupational factors (e.g., shifts, stress, violence, isolation) affect sleep, and that a reduction in the quantity or quality of sleep can have important consequences on the wellbeing, productivity, safety, and health of workers [53]. Sleep disorders are more frequent in older workers than in young employees. However, the medical surveillance of sleepiness and sleep disorders cannot be undertaken without the consent of the workers because this risk does not fall within those established by law.

Another example of the excessive divergence between the traditional risk prevention required by law and current reality concerns the police force. Police officers are known to be exposed to many occupational hazards, including psychosocial and musculoskeletal factors that are not listed among the “standard” ones. For this reason, only a part of the police force had the right to health surveillance which, in more than 70% of cases, was established for the risk of “work with video terminal display” [54]. In 2016, the Italian Society of Occupational Medicine produced Guidelines for the Surveillance of Police Forces [55], calling for the adoption of a proactive approach that would extend surveillance to all aspects of work and health including stress and cardiovascular risk [56]. On the basis of these indications, in 2017, the Chief of Police issued orders giving all police officers the possibility to undergo health checks and making health surveillance with evaluation of working capacity compulsory for older (>50 years) policemen and policewomen.

Countries such as the Netherlands and Germany, where the participatory process has already been adopted, are familiar with the difficulties involved. This process requires a large supply of readily available resources as well as the creation of networks of small and medium-sized enterprises coordinated by local organizations and supported by expertise from universities. Institutions that insure against occupational injuries should fund these efforts, and social partners should give their support.

Our analysis of experiences in EU countries showed that the successful development and implementation of WHPOW depends largely on involving both employees and management. Ageing is a highly complex phenomenon that requires a multi-level approach involving the collaboration of different departments in order to effectively manage the health of older workers. Activities undertaken as part of a wide-ranging strategy and implemented by large parent companies or local authorities are more likely to be sustained over time, and the presence of external consultants can offer valuable technical experience and expertise.

As resources are vital for sustaining WHPOW programs, it is essential to make both a preliminary assessment and regular evaluation of the measures adopted. Ideally, to be successful, a program should be based on a life-long approach, with actions that focus on introducing an early change in lifestyles for all employees. At the same time, it should also incorporate personalized and flexible measures. Intervention that includes flexible working hours, provision for mentoring or the transfer of knowledge, and extra days off would address some of the specific needs of the working population.

### 3.10. Type of Setting

Our study focused on intervention studies that were developed or implemented in the workplace [2,3,4,5,6]. The workplace is considered to be particularly interesting and important for health promotion because it has a powerful impact on a large number of people both during and after their working life. The development of WHP projects is facilitated in large enterprises because of the availability of medical resources and staff skilled in environmental and human resources management. Moreover, the fact that the workplace brings together large numbers of workers in a single place at a given time is an additional incentive for performing health promotion in that setting. Another vital aspect of health promotion in the workplace is the natural need for cooperation between employers and employees.

Nevertheless, the fact that health promotion action usually takes place in the workplace does not mean that all intervention studies adopt a “workplace setting approach,” i.e., all health promotion action begins and ends in the same environment. Poland et al. [57] stated that taking a “setting approach” to health promotion means not only addressing the contexts in which people live, work, and play and making these the object of inquiry and intervention, but it also involves considering the needs and capacities of people in different settings. The authors of this type of health promotion project recognize that both the problem and the solution lie within the same setting and that intervention must change the setting itself. In these projects, changing the central and underlying health factors does not merely involve changing people’s health behavior, it also entails modifying the settings themselves [58,59]. An example of an intervention study with a workplace setting approach is the participatory intervention described by Thorsen et al. [60] in which more fruit and vegetables were served in canteens, thus giving access to tasty and healthy food choices and reducing the availability of unhealthy options.

Rarely can the promotion of workers’ health be obtained only through changes in conditions in the workplace. When this occurs, rather than health promotion, it is often merely a question of preventing occupational risks, which, according to European Directives, is a mandatory task of the employer. We did not include such activities devoted exclusively to the safety and control of industrial risk factors in our research. Most health promotion needs to go beyond the boundaries of working activities by using a holistic approach to focus on physical, social and organizational factors. An example of a “non-settings” approach is when the workplace becomes a place where people can be contacted and their behavior can be modified in relation to diet, physical activity, or other non-occupational factors. In this way, health promotion activities can be performed in a setting without applying a “settings” approach.

Our review observed that the sole aim of most WHPOW intervention studies was to change the habits and lifestyles of workers and did not envisage any change in or control of the working environment. These studies adopted a “non-settings” approach as they used the workplace only as an environment in which health-promoting activities could be carried out on workers in order to change their behavior in relation to lifestyle factors such as diet, smoking and physical activity. They did not focus on the setting itself.

### 3.11. Type of Approach to Changes in the Environment

We also observed that health promotion projects with a “settings” approach to the workplace followed two distinct methods: a non-participatory, top-down approach, and a participatory, bottom-up approach. Participatory studies used group interventions that enabled workers to have a say in how the actions were conducted [61]. Examples included health circles, problem-based learning groups, and many others that required an active effort on the part of the employees. Most of these WHPOW studies used psychosocial measures as indicators since they wanted to change the organizational culture and climate toward older workers. Studies of this type used more qualitative research methods than those that adopted a different approach.

The non-participatory “settings” approach included studies that aimed at improving health by altering the physical work environment or work organization without involving employees in decisions about what changes should be made or how these should come about. These studies relied mainly on the opinions of experts and on technical measures, and often resorted to industrial hygiene or human resource management methods.

For some years now, the literature has indicated the participatory model as an example that should be followed for promoting health [62].

### 3.12. Concept of Health

Since the studies included in our selection did not explicitly define health, we studied the health outcomes resulting from intervention. An analysis of the intervention undertaken enabled us to deduce the underlying concept of health. According to Torp and Vinje [63], health promotion projects are based on three different concepts of health: the traditional idea that sees health as absence of disease (therefore health behavior, injury, occupational or non-occupational diseases, absenteeism), the salutogenic concept of positive health (i.e., engagement, satisfaction, self-esteem, motivation), and a middle-of-the-road definition or description of health based on dual intervention (work ability, general health).

The majority of studies in the workplace referred to pathogenic health outcomes such as the presence/absence of diseases or injury. Examples of these outcomes are common mental disorders, such as anxiety, depression, distress, or burnout; musculoskeletal disorders; cardiovascular diseases; allergy; and accidents. Many studies included health behavior action that focused on safe working techniques and the use of personal protective equipment at work. Some studies included lifestyle measures such as healthy eating, physical activity, and non-smoking behavior that were clearly detached from the core activities of the company. Health outcomes measured in other studies were absenteeism, i.e., the frequency of absence from work due to illness, or presenteeism, i.e., working despite being ill.

A more limited number of studies used outcomes such as general health and the quality of life or work ability, often measured by the Finnish Work Ability Index. Both the quality of life and work ability can be used as positive measurements, but they are used more frequently for the negative measurement of injuries or work-related diseases.

Positive health outcomes such as self-esteem and job engagement were rarely considered. Most studies used more than one outcome measurement related to health. Moreover, several studies included outcome measurements related to the working environment, productivity and satisfaction with the type of intervention.

## 4. Conclusions

This review of action carried out in the workplace to promote healthy ageing found that the principal obstacles to be overcome are deficiencies and inflexibility. The lack of economic and human resources is a hindrance, especially in small businesses, but a much greater obstacle is the lack of attention given to the problem by company managers who fail to understand the importance of activating a virtuous cycle through investment in health promotion. The inflexibility of older workers who insist on maintaining living and working habits also represents a barrier to health promotion, but what causes greater difficulty is the intractable approach of OHS systems that are oriented more toward the prevention of traditional risks than the promotion of a state of full health and wellbeing.

An assessment of the effectiveness of WHPOW programs might help management understand the importance of health actions. It is therefore advisable to give maximum dissemination to effectiveness studies and publish negative results so that others can identify errors and avoid repeating them.

Although the literature indicates that health promotion interventions based on a salutogenic approach and a participatory method designed to modify lifestyles and work environments are the most effective, the majority of studies we surveyed adopted a traditional clinical approach focused mainly outside the workplace. Any changes that were made to the place of work were almost always decided with a top-down approach, whereas it would be desirable to use a participatory, bottom-up, and salutogenic approach to conduct multi-level interventions directed at both the working and living environment.

Occupational health promotion is linked to at least two more strategies for improving health in the workplace: (1) wellness programs, with emphasis on the lifestyle of individuals; and (2) occupational health and safety with a focus on physical work-related risk factors [64]. Most experts [18,65] argue that WHP should adopt a holistic approach that includes both of these aspects and should also focus on psychosocial and organizational work factors. Health promotion interventions should be designed to bring about structural changes in production that would significantly improve the work situation. This general principle applies to health promotion as a whole, but it is of particular importance when it comes to older workers who, due to their physical and mental characteristics, have greater difficulty than young people in adapting to difficult working conditions.

There are three main approaches to health promotion that place a focus on (1) a problem (e.g., accident prevention and reducing smoking levels); (2) the population (e.g., ageing in good health); or (3) the setting (e.g., schools that promote health). Most studies conducted in the workplace are designed to alter health behavior via targeted intervention rather than to change the work environment. However, this type of approach is not the most successful when dealing with older workers who are generally more rooted in their life and work habits than young people.

Despite the large amount of theoretical health promotion literature that explicitly describes health as a positive concept related to physical, mental, and social well-being and not simply to the absence of disease, very few health promotion studies in the workplace use positive health measurements.

Both the Ottawa Charter for Health Promotion and the Luxembourg Declaration on Workplace Health Promotion in the European Union reflect a holistic view of health and explicitly refer to the WHO’s definition of health as a state of overall wellbeing. Wellbeing at work is closely related to positive aspects such as motivation, engagement, and job satisfaction [66]. The most recent literature emphasizes the idea that health must be seen as the ability to adapt and self-manage in the face of physical, social and emotional illnesses or constraints that are more or less chronic [67]. In accordance with Antonovsky’s [68] salutogenic model, individuals should be helped to move toward higher levels of overall health, wellbeing, and achievement. This positive, holistic, and dynamic approach emphasizes the need to focus on people’s resources and their capacity to create health [69].

Compared to practitioners in other medical fields, occupational physicians have always focused less on treatment and more on the prevention of accidents and diseases. The occupational health focus has been, and still is, mainly on risk factors and the prevention of diseases rather than on health promotion based on a positive and/or holistic approach. In European countries, research on workplace health promotion often resembles traditional disease prevention. In future, health promotion programs conducted in the workplace should focus on risk factors and the prevention of disease and should also stress the importance of a participatory approach directed toward positive health outcomes.

A gradual legislative alignment with the principles of health promotion is desirable in all European countries. Governments should aim to establish a better link between work and health because quality production cannot be obtained without increasing the quality of life. Resources should be recovered so as to avoid health consumerism in the workplace, and the authorities should promote the rehabilitation and recovery of older workers with chronic disorders rather than investing in compensation or pensions. Finally, social partners should be granted autonomy in health and safety in the workplace.

The ageing of the active population means that health promotion is a necessity rather than a mere option. European institutions should therefore urge national authorities to introduce further health promotion intervention, engage social partners and private bodies in this objective, and share experiences and the results of effectiveness tests.

## References

[1] Sitko S.J., Kowalska-Bobko I., Mokrzycka A., Zabdyr-Jamróz M., Domagała A., Magnavita N., Poscia A., Rogala M., Szetela A., Golinowska S. (2016). Institutional analysis of health promotion for older people in Europe. Concept and research tool. BMC Health Serv. Res..

[2] Magnavita N., Capitanelli I., La Milia D.I., Moscato U., Poscia A., Ricciardi W. (2017). Workplace health promotion programs in different areas of Europe. Epidemiol. Biostat. Public Health.

[3] Magnavita N., Capitanelli I., Lops E.A., La Milia D.I., Manetta S., Moscato U., Poscia A., Ricciardi W. (2017). Workplace health promotion in Europe. Findings from ProHealth65+. Eur. J. Public Health.

[4] Magnavita N. (2018). Health Promotion for Older Workers. European Experiences. Promuovere la Salute dei Lavoratori Anziani. Le Esperienze Europee.

[5] Poscia A., Moscato U., La Milia D.I., Milovanovic S., Stojanovic J., Borghini A., Collamati A., Ricciardi W., Magnavita N. (2016). Workplace Health Promotion for Older Workers: A Systematic Literature Review. BMC Health Serv. Res..

[6] Borghini A., Poscia A., La Milia D.I., Stojanovic J., Pattavina F., Tamburrano A., Ricciardi W., Moscato U., Magnavita N. (2016). Institutional analysis of workplace health promotion toward elderly in 10 European countries: The Pro-Health65+ project. Eur. J. Public Health.

[7] Tryon K., Bolnick H., Pomeranz J.L., Pronk N., Yach D. (2014). Making the workplace a more effective site for prevention of noncommunicable diseases in adults. J. Occup. Environ. Med..

[8] Watkins C., Macy G., Golla V., Lartey G., Basham J. (2017). The “Total Worker Health” Concept: A Case Study in A Rural Workplace. J. Occup. Environ. Med..

[9] Chong J., Ingram M., Mcclelland D.J., Lopez D.C., De Zapien J.G. (2000). Smoking behavior in a smoking workplace. J. Subst. Abuse.

[10] Nowalk M.P., Lin C.J., Toback S.L., Rousculp M.D., Eby C., Raymund M., Zimmerman R.K. (2010). Improving influenza vaccination rates in the workplace: A randomized trial. Am. J. Prev. Med..

[11] Shaw W.S., Besen E., Pransky G., Boot C.R., Nicholas M.K., McLellan R.K., Tveito T.H. (2014). Manage at work: A randomized, controlled trial of a self-management group intervention to overcome workplace challenges associated with chronic physical health conditions. BMC Public Health.

[12] Wierenga D., Engbers L.H., Van Empelen P., Duijts S., Hildebrandt V.H., Van Mechelen W. (2013). What is actually measured in process evaluations for worksite health promotion programs: A systematic review. BMC Public Health.

[13] Van Dalen H.P., Henkens K., Wang M. (2015). Recharging or Retiring Older Workers? Uncovering the Age-Based Strategies of European Employers. Gerontologist.

[14] International Labour Organization (ILO) (2012). SOLVE: Integrating Health Promotion into Workplace OSH Policies.

[15] Stephens S.K., Cobiac L.J., Veerman J.L. (2014). Improving diet and physical activity to reduce population prevalence of overweight and obesity: An overview of current evidence. Prev. Med..

[16] Janecka I. (2017). Health, Health Care, and Systems Science: Emerging Paradigm. Cureus.

[17] Harris J.R., Hannon P.A., Beresford S.A., Linnan L.A., McLellan D.L. (2014). Health promotion in smaller workplaces in the United States. Annu. Rev. Public Health.

[18] Torp S., Eklund L., Thorpenberg S. (2011). Research on workplace health promotion in the Nordic countries—A literature review; 1986–2008. Glob. Health Promot..

[19] Bulotaitė L., Šorytė D., Vičaitė S., Šidagytė R., Lakiša S., Vanadziņš I., Kozlova L., Eglīte M., Hopsu L., Salmi A. (2017). Workplace health promotion in health care settings in Finland, Latvia, and Lithuania. Medicina.

[20] Vanhoorne M.N., Vanachter O.V., De Ridder M.P. (2006). Occupational health care for the 21st century: From health at work to workers’ health. Int. J. Occup. Environ. Health.

[21] Hinrichs S., Bromberg T., Fries-Tersch E. (2016). Safer and Healthier Work at Any Age Country Inventory: Germany. https://osha.europa.eu/en/tools-and-publications/publications/safer-and-healthier-work-any-age-country-inventory-germany/view.

[22] Skriabikova O.J., Kuipers Cavaco Y.M., Fries-Tersch E. (2016). Safer and Healthier Work at Any Age. Country Inventory: The Netherlands. https://osha.europa.eu/en/tools-and-publications/publications/safer-and-healthier-work-any-age-country-inventory-netherlands/view.

[23] Schröer S., Haupt J., Pieper C. (2014). Evidence-based lifestyle interventions in the workplace—An overview. Occup. Med. (Lond.).

[24] Manzoli L., Sotgiu G., Magnavita N., Durando P., National Working Group on Occupational Hygiene of the Italian Society of Hygiene Preventive Medicine and Public Health SItI (2015). Evidence-based approach for continuous improvement of occupational health. Epidemiol. Prev..

[25] Palmer K.T., Harris E.C., Linaker C., Barker M., Lawrence W., Cooper C., Coggon D. (2012). Effectiveness of community- and workplace-based interventions to manage musculoskeletal-related sickness absence and job loss: A systematic review. Rheumatology.

[26] Harris J.R., Cheadle A., Hannon P.A., Forehand M., Lichiello P., Mahoney E., Snyder S., Yarrow J. (2012). A framework for disseminating evidence-based health promotion practices. Prev. Chronic Dis..

[27] Rees D., Phillips J.I. (2014). Investigating the effectiveness of occupational health interventions in the workplace. Occup. Environ. Med..

[28] Cremaschini M., Moretti R., Valoti M., Barbaglio G., Arnoldi M., Bigoni F., Guaschi E., Merisi F., Passera P., Rebba A. (2017). Delphi consensus on the monitoring tools of main individual risk factors for chronic diseases by the company doctor. Med. Lav..

[29] Astrella J.A. (2017). Return on Investment: Evaluating the Evidence Regarding Financial Outcomes of Workplace Wellness Programs. J. Nurs. Adm..

[30] Tam G., Yeung M.P.S. (2017). A systematic review of the long-term effectiveness of work-based lifestyle interventions to tackle overweight and obesity. Prev. Med..

[31] Allan J., Querstret D., Banas K., de Bruin M. (2017). Environmental interventions for altering eating behaviours of employees in the workplace: A systematic review. Obes. Rev..

[32] Weerasekara Y.K., Roberts S.B., Kahn M.A., LaVertu A.E., Hoffman B., Das S.K. (2016). Effectiveness of Workplace Weight Management Interventions: A Systematic Review. Curr. Obes. Rep..

[33] Brinkley A., McDermott H., Munir F. (2017). What benefits does team sport hold for the workplace? A systematic review. J. Sports Sci..

[34] Joyce S., Modini M., Christensen H., Mykletun A., Bryant R., Mitchell P.B., Harvey S.B. (2016). Workplace interventions for common mental disorders: A systematic meta-review. Psychol. Med..

[35] Rongen A., Robroek S.J., van Lenthe F.J., Burdorf A. (2013). Workplace health promotion: A meta-analysis of effectiveness. Am. J. Prev. Med..

[36] Pereira M.J., Coombes B.K., Comans T.A., Johnston V. (2015). The impact of onsite workplace health-enhancing physical activity interventions on worker productivity: A systematic review. Occup. Environ. Med..

[37] Lerner D., Rodday A.M., Cohen J.T., Rogers W.H. (2013). A systematic review of the evidence concerning the economic impact of employee-focused health promotion and wellness programs. J. Occup. Environ. Med..

[38] Osilla K.C., Van Busum K., Schnyer C., Larkin J.W., Eibner C., Mattke S. (2012). Systematic review of the impact of worksite wellness programs. Am. J. Manag. Care.

[39] Maes L., Van Cauwenberghe E., Van Lippevelde W., Spittaels H., De Pauw E., Oppert J.M., Van Lenthe F.J., Brug J., De Bourdeaudhuij I. (2012). Effectiveness of workplace interventions in Europe promoting healthy eating: A systematic review. Eur. J. Public Health.

[40] Cancelliere C., Cassidy J.D., Ammendolia C., Côté P. (2011). Are workplace health promotion programs effective at improving presenteeism in workers? A systematic review and best evidence synthesis of the literature. BMC Public Health.

[41] Cherniack M. (2013). Integrated health programs; health outcomes, and return on investment: Measuring workplace health promotion and integrated program effectiveness. J. Occup. Environ. Med..

[42] Goetzel R.Z., Henke R.M., Tabrizi M., Pelletier K.R., Loeppke R., Ballard D.W., Grossmeier J., Anderson D.R., Yach D., Kelly R.K. (2014). Do workplace health promotion (wellness) programs work?. J. Occup. Environ. Med..

[43] Kim Y., Park J., Park M. (2016). Creating a Culture of Prevention in Occupational Safety and Health Practice. Saf. Health Work.

[44] Pinkstaff S.O., McNeil A., Arena R., Cahalin L. (2017). Healthy Living Medicine in the Workplace: More Work to Do. Prog. Cardiovasc. Dis..

[45] Dobras M., Sakowski P., Fries-Tersch E. (2016). Safer and Healthier Work at Any Age Country Inventory: Poland. https://osha.europa.eu/en/tools-and-publications/publications/safer-and-healthier-work-any-age-country-inventory-poland/view.

[46] Baldasseroni A., Bernhardt S., Cervino D., Gardini A., Salizzato L. (2004). Dossier SALeM: A method for assessing the effectiveness of a public health program. Epidemiol. Prev..

[47] Magnavita N., Baldasseroni A., Santoro P.E., Magnavita G., Bergamaschi A. (2010). Admission to medical graduate programs in Italian universities. Inequality and discrimination. Giornale italiano di medicina del lavoro ed ergonomia..

[48] Meng L., Wolff M.B., Mattick K.A., DeJoy D.M., Wilson M.G., Smith M.L. (2017). Strategies for Worksite Health Interventions to Employees with Elevated Risk of Chronic Diseases. Saf. Health Work.

[49] Magnavita N., Capitanelli I., Garbarino S., La Milia D.I., Moscato U., Pira E., Poscia A., Ricciardi W. (2017). Workplace health promotion programs for older workers in Italy. Med. Lav..

[50] Pachman J. (2009). Evidence base for pre-employment medical screening. Bull. World Health Organ..

[51] Pronk N.P. (2013). Integrated worker health protection and promotion programs: Overview and perspectives on health and economic outcomes. J. Occup. Environ. Med..

[52] Garbarino S., Magnavita N. (2014). Obstructive Sleep Apnea Syndrome (OSAS); metabolic syndrome and mental health in small enterprise workers. Feasibility of an Action for Health. PLoS ONE.

[53] Magnavita N., Garbarino S. (2017). Sleep, Health and Wellness at Work: A Scoping Review. Int. J. Environ. Res. Public Health.

[54] Pira E., Garbarino S., Ciprani F., De Lorenzo G., Mennoia N.V., Proto E., Roca A., Magnavita N. (2016). Guidelines on health surveillance of Italian Police Forces: An empty space to fill. Med. Lav..

[55] Pira E., Garbarino S., Magnavita N., Ciprani F., De Lorenzo G., Garzaro G., Mennoia N.V., Proto E., Roca A. (2016). Guidelines for the Health Surveillance of operators of the Police Corps.

[56] Magnavita N., Capitanelli I., Garbarino S., Pira E. (2018). Work-related stress as a cardiovascular risk factor in police officers. A systematic review of evidence. Int. Arch. Occup. Environ. Health.

[57] Poland B., Krup G., McCall D. (2009). Settings for health promotion: An analytic framework to guide intervention design and implementation. Health Promot. Pract..

[58] Whitelaw S., Baxendale A., Bryce C., MacHardy L., Young I., Witney E. (2001). “Settings” based health promotion: A review. Health Promot. Int..

[59] Dooris M. (2009). Holistic and sustainable health improvement: The contribution of the settings-based approach to health promotion. Perspect. Publ. Health.

[60] Thorsen A.V., Lassen A.D., Tetens I., Hels O., Mikkelsen B.E. (2010). Long-term sustainability of a worksite canteen intervention of serving more fruit and vegetables. Public Health Nutr..

[61] Siedlecka J., Gadzicka E., Szyjkowska A., Siedlecki P., Szymczak W., Makowiec-Dąbrowska T., Bortkiewicz A. (2017). Prevention of cardiovascular diseases—Prophylactic program in a selected enterprise. Med. Pr..

[62] Punnett L., Warren N., Henning R., Nobrega S., Cherniack M., CPH-NEW Research Team (2013). Participatory ergonomics as a model for integrated programs to prevent chronic disease. J. Occup. Environ. Med..

[63] Torp S., Vinje H.F. (2014). Is workplace health promotion research in the Nordic countries really on the right track?. Scand. J. Public Health.

[64] Chu C., Breucker G., Harris N., Stitzel A., Gan X., Gu X., Dwyer S. (2000). Health promoting workplaces—International settings development. Health Prom. Int..

[65] Shain M., Kramer D.M. (2004). Health promotion in the workplace: Framing the concept; reviewing the evidence. Occup. Environ. Med..

[66] Magnavita N. (2017). Productive aging; work engagement and participation of older workers. A triadic approach to health and safety in the workplace. Epidemiol. Biostat. Public Health.

[67] Huber M., Knottnerus J.A., Green L., van der Horst H., Jadad A.R., Kromhout D., Leonard B., Lorig K., Loureiro M.I., van der Meer J.W. (2011). How should we define health?. BMJ.

[68] Antonovsky A. (1996). The salutogenic model as a theory to guide health promotion. Health Promot. Int..

[69] Mittelmark M.B., Sagy S., Eriksson M., Bauer G.F., Pelikan J.M., Lindström B., Espnes G.A. (2017). The Handbook of Salutogenesis.

